# Proteomic profiling of HBV infected liver biopsies with different fibrotic stages

**DOI:** 10.1186/s12953-017-0114-4

**Published:** 2017-04-20

**Authors:** Seyma Katrinli, Kamil Ozdil, Abdurrahman Sahin, Oguzhan Ozturk, Gozde Kir, Ahmet Tarik Baykal, Emel Akgun, Omer Sinan Sarac, Mehmet Sokmen, H. Levent Doğanay, Gizem Dinler Doğanay

**Affiliations:** 1 Molecular Biology Biotechnology and Genetics Research Center (MOBGAM), Istanbul Technical University, Sariyer, Istanbul, Turkey; 2 Gastroenterology, Umraniye Teaching and Research Hospital, Umraniye, Istanbul, Turkey; 3 Pathology, Umraniye Teaching and Research Hospital, Umraniye, Istanbul, Turkey; 4 Department of Medical Biochemistry, School of Medicine, Acibadem University, Istanbul, Turkey; 5 Computer Engineering, Istanbul Technical University, Sarıyer, Istanbul, Turkey

**Keywords:** Liver fibrosis, Chronic hepatitis B, Two-dimensional difference gel electrophoresis, Proteomics, Glycolysis

## Abstract

**Background:**

Hepatitis B virus (HBV) is a global health problem, and infected patients if left untreated may develop cirrhosis and eventually hepatocellular carcinoma. This study aims to enlighten pathways associated with HBV related liver fibrosis for delineation of potential new therapeutic targets and biomarkers.

**Methods:**

Tissue samples from 47 HBV infected patients with different fibrotic stages (F1 to F6) were enrolled for 2D-DIGE proteomic screening. Differentially expressed proteins were identified by mass spectrometry and verified by western blotting. Functional proteomic associations were analyzed by EnrichNet application.

**Results:**

Fibrotic stage variations were observed for apolipoprotein A1 (APOA1), pyruvate kinase PKM (KPYM), glyceraldehyde 3-phospahate dehydrogenase (GAPDH), glutamate dehydrogenase (DHE3), aldehyde dehydrogenase (ALDH2), alcohol dehydrogenase (ALDH1A1), transferrin (TRFE), peroxiredoxin 3 (PRDX3), phenazine biosynthesis-like domain-containing protein (PBLD), immuglobulin kappa chain C region (IGKC), annexin A4 (ANXA4), keratin 5 (KRT5). Enrichment analysis with Reactome and Kegg databases highlighted the possible involvement of platelet release, glycolysis and HDL mediated lipid transport pathways. Moreover, string analysis revealed that HIF-1α (Hypoxia-inducible factor 1-alpha), one of the interacting partners of HBx (Hepatitis B X protein), may play a role in the altered glycolytic response and oxidative stress observed in liver fibrosis.

**Conclusions:**

To our knowledge, this is the first protomic research that studies HBV infected fibrotic human liver tissues to investigate alterations in protein levels and affected pathways among different fibrotic stages. Observed changes in the glycolytic pathway caused by HBx presence and therefore its interactions with HIF-1α can be a target pathway for novel therapeutic purposes.

**Electronic supplementary material:**

The online version of this article (doi:10.1186/s12953-017-0114-4) contains supplementary material, which is available to authorized users.

## Background

Chronic hepatitis B (CHB) is a global health care problem, around two billion people have been infected with the virus and annually 800.000 people lose their lives due to consequences of hepatitis B infection [1]. The virus itself is non-cytopathic, and ongoing inappropriate inflammatory response against hepatitis B virus (HBV) causes the liver damage [2]. Inflammation in liver triggers activation of effector cells, mainly hepatic stellate cells, and this yields to deposition of extracellular matrix causing fibrosis [3]. Approximately out of 10% of HBV infected patients, this ongoing fibrogenesis culminates in cirrhosis [4] and, if those with cirrhosis left untreated, the five-year survival drops to 50% [5]. Furthermore, annually 2% of cirrhotic patients develops hepatocellular carcinoma (HCC) and in total half of all liver cancers are due to HBV, worldwide [1, 6]. To estimate which patient with CHB will develop fibrosis and/or cirrhosis rapidly, is yet a controversial issue. Although many pathological and epidemiological research have suggested that several elements, including host genetic factors, viral factors, inflammation and alcohol consumption are involved in the development of fibrosis in chronic HBV, hepatic cellular pathways causing liver fibrosis in HBV infection is not yet fully understood [7–10]. Advances in molecular and biochemical technologies are expected to clarify the pathways involved in development of HBV associated liver fibrosis, and efforts to do so might yield to the discovery of potential targets for novel anti-fibrotic treatments.

Proteomic studies on liver fibrosis mainly focused on HCC or hepatitis C driven cirrhosis with limited samples. One of the earlier studies has compared tumor tissue and surrounding non-tumor tissue from eight HCC patients and has showed overexpression of 14-3-3ɣ protein in HCC [11]. Another study investigated the proteomic differences between tumor and adjacent nontumor tissue samples of 12 HBV-associated HCC patients and found out upregulation of members of the heat shock protein 70 and 90 families and downregulation of metabolism-associated and mitochondrial and peroxisomal proteins in HCC [12]. Molleken et al., also has analyzed cirrhotic septa and liver parenchyme of seven cirrhotic patients and has discovered an increase in cell structure associated proteins which are actin, prolyl 4-hydroxylase, tropomyosin, calponin, transgelin and human microfibril-associated protein 4 (MFAP-4) [13]. However, all these studies investigate the alterations occuring at the very late stage of fibrosis and did not give information about the proteomic changes during fibrosis progression. To understand the pathways related to fibrosis, the proteomic changes between different fibrotic stages should be investigated. Proteomic studies evaluating serum or plasma proteins corresponding to different fibrotic stages of HCV and HBV infected patients exist in literature [14–17]. Although these studies are promising for determining serum biomarkers for non-invasive diagnosis of liver fibrosis, they are insufficient for exposing pathways related to fibrosis progression. Such a study should involve analysis of liver tissue samples with different fibrotic stages and so far, there are a few research available. In one of these research, HCV-infected human liver tissue from 15 patients at different stages of fibrosis were analyzed by ^16^O/^18^O stable isotope labeling in combination with the accurate mass and time (AMT) tag approach and demonstrated association of oxidative stress and hepatic mitochondrial dysfunction with HCV pathogenesis [18]. A recent study performed differential label-free proteomics approach using 27 biopsies from patients with HCV-associated hepatic fibrosis and reported alterations in lumican (LUM), fibulin 5 (FBLN5), cysteine and glycine-rich protein 2 (CSRP2), calponin 2 (CNN2), transgelin (TAGLN), collagen alpha-1(XIV) chain (COL14A1), and MFAP-4, then verify the expression of these proteins on a transcriptional level and with targeted proteomic approach in different cohorts composed by a total of 77 and 68 HBV or HCV infected patients with liver fibrosis, respectively [19]. These mentioned studies focuses on proteomics of HCV associated liver fibrosis, only the latter offered a partial targeted proteomic information about limited group corresponding to different fibrotic stages in HBV infection. Hereby, a broad fibrotic stage specific proteomic profiling study of HBV associated liver fibrosis is still missing in literature.

In this study, to obtain an insight into the pathogenesis of HBV related liver fibrosis, we firstly performed differential proteome analysis of the human liver tissue specimens with different fibrotic stages classified according to Ishak, using two dimensional difference gel electrophoresis (2D-DIGE) followed by tandem mass spectrometry. Findings of this study will highlight cellular pathways associated with liver fibrosis in HBV infection for future development of new treatments through detection of target proteins for drug development, and will offer putative stage specific biomarkers.

## Methods

### Patients

Human liver tissues from 47 chronic HBV patients were collected between 2014 and 2015 from Gastroenterology and Hepatology Clinic at Umraniye Teaching and Research Hospital with the prior approval of the Ethical Committee at the teaching hospital and with prior written informed consents obtained from patients. All patients had been diagnosed CHB and were followed up at the hepatology clinic. All were HBsAg positive with detectable HBV-DNA. All had been considered as a candidate for antiviral treatment and had been scheduled for liver biopsy. Patients co-infected with other hepatitis virus (hepatitis delta virus, HCV) or HIV infection, patients with other liver diseases (hemochromatosis, alpha 1 anti-trypsin deficiency, auto immune hepatitis, primary biliary cirrhosis, sclerosing cholangitis, Wilson’s disease, obstructing biliary disease, malignancy, non-alcoholic steatohepatitis) or patients with alcohol intake more than 20 mg/day or patients taking antiviral treatment or immunosuppressive treatment were all excluded. All tissues were obtained before antiviral therapy. Biopsies were obtained under ultrasonographic guidance by 16G Hepafix needle. The length of the biopsies was not shorter than 2.5 cm. All biopsy specimens were stained with haematoxyin-eosin and Masson’s trichrome. Classification of the fibrotic stages and inflammation grades were done according to Ishak’s classification by an experienced pathologist who was blinded to proteomic analysis [20].

The study was conducted in accord with the ethical principles originating in the Declaration of Helsinki.

### Sample preparation of tissue samples

Fresh frozen liver tissue specimens were thawed and each were homogenized with 120 μL of T-PER™ Tissue Protein Extraction Reagent (Pierce Chemical, Rockford, IL) containing 0,1% (v/v) protease inhibitor cocktail (Roche, Mannheim, Germany) using hand homogenizer. The homogenates were incubated 5 min on ice and then centrifuged at 10.000×g for 5 min. The resulting supernatant was collected, and the protein concentration was measured by BCA assay with BSA as the standard. The ReadyPrep™ 2-D Cleanup Kit (Bio-Rad, Hercules, CA, USA) was further used to purify the homogenate according to the manufacturer’s protocol. Before processing to 2D-DIGE, samples in the same group were pooled to decrease individual differences.

### Two dimension-difference gel electrophoresis (2D-DIGE)

For tissue samples, the same group of sample was labelled with either Cy3 or Cy5 and ran in two different gels to eliminate the effect of dyes. 60 μg protein of each group was labelled with 400 pmol of Cy3 or Cy5 and 60 μg protein from internal standard (10 μg from each group) was labelled with Cy2. After 30 min incubation in dark, the labeling reaction was stopped by adding 1 μL of 10 mM lysine. Two different groups (Cy3 and Cy5) and the internal standard (Cy2) were run per gel. The three labelled samples were mixed and the volume was adjusted to 150 μL with rehydration buffer (8 M urea, 2% CHAPS, 50 mM dithiothreitol (DTT), 0.2% (w/v) Bio-Lyte® 3/10 ampholytes, and trace amount of Bromophenol Blue). All gels, ten in total, were processed and analyzed simultaneously. The first dimension was performed on Protean IEF Cell (Bio-Rad) using 7 cm, pH 5-8 IPG gel strips. Isoelectric focusing (IEF) was carried on at 20 °C under the following conditions: 14 h at 50 V; 20 min at 250 V; 2 h at 4000 V and held at 4000 V until total Vh reached 20000 Vh. After IEF, the IPG strips were equilibrated for 15 min in a reduction buffer (6 M urea, 2% SDS, 0.375 M Tris-HCl (pH 8.8), 20% glycerol, and 2% (w/v) DTT) and subsequently alkylated for 10 min in an alkylation buffer (6 M urea, 2% SDS, 0.375 M Tris-HCl (pH 8.8), 20% glycerol and 2.5% iodoacetamide). After equilibration, the strips were overlaid on individual 12% polyacrylamide gels and added 0.5% agarose to immobilize the strips. The second dimensional separation was carried out in the Bio-Rad Mini Protean system and the electrophoresis was run at 125 V for 1.5 h.

### Gel image and data analysis

The gels were scanned using ChemiDoc™ MP Imaging System (Bio-Rad) at three different settings (Cy2, blue laser 488 nm and 520 bp 40 filter; Cy3, green laser 532 nm and 580 bp 30 filter; Cy5, red laser 633 nm and 670 bp 30 filter). The scanned gels were semi-automatically matched and analyzed by the PD-Quest software v.8.0.1 (Bio-Rad). Spot volumes (pixels*spot size) were assigned to each protein spot and normalized to internal standard. In our experimental design, besides Cy2 labeled internal pooled standard, we also run Cy3 or Cy5 labeled F1 pool in each gel. Therefore, we had two internal controls which enabled better alignment of gels for more accurate spot analysis. For proteome profile comparison, proteome profile of each six Ishak fibrotic stage (F1-F6) was compared to each other by Student’s *t*-test in search for differentially expressed spots between stages. The quantitative difference in percent volume of spots more than two fold was considered as differential expression change. Competent spots with a p-value ≤0.05 were considered statistically significant and withheld for protein identification by mass spectrometry.

### In-gel digestion and mass spectrometric analysis

Spot picking was carried out with preparative gels. Two-dimensional electrophoresis was performed as described under “Two dimension-difference gel electrophoresis” except that the IPG strips were loaded with 300 μg of protein consisting of equal amounts from each stage, and gels were stained with colloidal Coomassie Brillant Blue. Protein spots of interest were matched between the images of analytical and preparative gels and manually cut from preparative gels; then and transferred to a 1.5 mL siliconikzed Eppendorf tube. Subsequently, the transferred gel spots were destained in a destaining solution (100 μL acetonitrile (ACN): 100 mM ammonium bicarbonate (ABC), v/v, 1:1). After prereduction at 50 °C using 100% acetonitrile, gel pieces were reduced (10 mM DTT at 80 °C for 30 min) and then alkylated (20 mM iodoacetamide at room temperature and dark for 45 min). After a subsequent washing step (250 mM ABC and 50% ACN), the pieces were dehydrated in 100% ACN and dried in at 50 °C. A total volume of 30 μL trypsin (20 ng/μL in 50 mM ABC) added to the samples incubated overnight at 37 °C.

Peptide samples were dissolved in 1% formic acid and 2% ACN, then peptide sequences were analysed by tandem mass spectrometry (MS/MS) on a SYNAPT G2-Si mass spectrometer (Waters, Milford, MA, USA). Peak list files containing mass spectral data were processed using the ProteinLynxGlobalServer software (PLGS V3.02; Waters) and were searched against the reviewed Uniprot homosapiens database. Database search parameters were set according to previously published data [21].

### Validation with western blotting

Fifty microgram of tissue proteins from each fibrotic stage pool were mixed with NuPAGE® LDS Sample Buffer (4X) and NuPAGE® Reducing Agent (10X) (Invitrogen, CA, USA) and were heated at 95 °C for 5 min, prior to loading to 12% sodium dodecyl sulfate-polyacrylamide gel electrophoresis (SDS-PAGE). The iblot2 system (Invitrogen, CA, USA) was used for transferring the proteins to polyvinylidene fluoride (PVDF) membrane. After using 0.5% skim milk to blot the membrane for 2 h at room temperature, APOA11, KPYM, GAPDH, DHE3, ALDH1, ALDH1A1, TRFE, PRDX3, PBLD were detected by mouse anti-APOA11 antibody (Cell Signaling Technology, CST, 1:250), rabbit anti-KPYM antibody (CST, 1:250), rabbit anti-GAPDH antibody (CST, 1:1000), rabbit anti-DHE3 antibody (CST, 1:500), mouse anti-ALDH2 antibody (Thermo Scientific, 1:500), rabbit anti-ALDH1A1 antibody (CST, 1:500), mouse anti-TRFE (Thermo Scientific, 1:500), rabbit anti-PRDX3 (Thermo Scientific, 1:250), mouse anti-PBLD (Novus, 1:1000) at 4 °C overnight. After washing three times in TBS-T buffer (137 mM Sodium Chloride, 20 mM Tris, 0.1% Tween-20, pH 7.6), the second antibody conjugated with horseradish peroxidase [anti-mouse (CST, 1:5000) or anti-rabbit (CST, 1:5000), depending on applied primer antibody]. Protein signals were detected by ECL detection system (Bio-Rad, Hercules, CA, USA) and images were acquired by ChemiDoc™ MP Imaging System (Bio-Rad). Quantitative analysis of western blotting images was performed by ImageLab software v5.2.1 (Bio-Rad) and statistical analysis was done by Student’s *t*-test. Normalization could not be done by a cytosolic housekeeping protein (i.e. β-actin, β-tubulin, GAPDH) due to their expressional changes within the different fibrotic stages, therefore, both rabbit anti-histoneH3 antibody (CST, 1:500) and ponceau statining were used for loading control and normalization.

### Associations between identified proteins and cellular pathways in liver

Functional associations between identified proteins from liver tissue and cellular pathways were analyzed by processing the protein list with on-line EnrichNet application [22] using KEGG [23] and Reactome [24] databases. The significance of overlap between protein sets was decided by using a combination of one-side Fisher’s exact test (*q* < 0.05) and network similarity scores (XD-scores). The threshold values were assessed by EnrichNet with a regression fit equivalent to a Fisher q value of 0.05 and an upper boundary of 95% confidence for linear fitting. The trend of these identified pathways among development of fibrotic stages was determined by Reactome expression analysis. Interation networks for the differentially expressed genes were conducted using the STRING tool [25].

## Results and discussion

### Selection of study group

Human liver tissues from 47 chronic HBV patients [male = 30, female = 17; mean age (range) = 41.3 (19–65) years] were collected between 2014 and 2015 from Gastroenterology and Hepatology Clinic at Umraniye Teaching and Research Hospital. Fibrosis stages according to Ishak F-score of these 47 subjects were as followed: F1 (*n* = 7), F2 (*n* = 20), F3 (*n* = 12), F4 (*n* = 3), F5 (*n* = 2), F6 (*n* = 3). In addition, patient’s characteristics and laboratory results within 10 days of biopsy were given on Table 1. A more detailed demographic data of patient’s according to fibrotic stages is also presented in Additional file 1: Table S1.

**Table 1:** Patient’s characteristics of the proteome study (*n* = 47)

		Statistics (*N* = 47)
Age (mean ± sem)		40.98 ± 2.063 (19–66)
Sex	Male	30 (63.8%)
	Female	17 (36.2%)
F-score	1	7
	2	20
	3	12
	4	3
	5	2
	6	3
HAI (mean ± sem)		5.36 ± 0.366 (2–11)
logHBVDNA (mean ± sem)		6.04 ± 0.263 (1.77–8.94)
AST (mean ± sem)		54.59 ± 7.697 (16–309)
ALT (mean ± sem)		88.89 ± 14.569 (14–441)
PLT (x10^3^) (mean ± sem)		218 ± 8.4 (119–370)
PT (mean ± sem)		13.23 ± 0.13 (11.4–16.1)
INR (mean ± sem)		1.03 ± 0.0178 (0.85–1.39)
HDL (mean ± sem)		46.65 ± 2.29 (27–72)
LDL (mean ± sem)		116 ± 5.27 (60–167)
Triglyceride (mean ± sem)		105.17 ± 9.59 (36–359)
Glucose (mean ± sem)		103.02 ± 6.1 (76–295)
Waist (cm) (mean ± sem)		92.48 ± 2.1 (62–120)
BMI (mean ± sem)		27 ± 0.7 (19–38)
HBeAg	Positive	11 (23.4%)
	Negative	36 (76.6%)

*SEM* standard error of mean, *F* fibrosis, *HAI* hepatic activity index, *HBV* hepatitis B virus, *AST* aspartate transaminase, *ALT* alanine transaminase, *PLT* platelet, *PT* prothrombin time, *INR* international normalized ratio, *HDL* high density lipoprotein, *LDL* low density lipoprotein, *BMI* body mass index, *HBeAg* hepatitis B early antigen

### Quantitative comparison and identification of protein spots

**Fig. 1:**
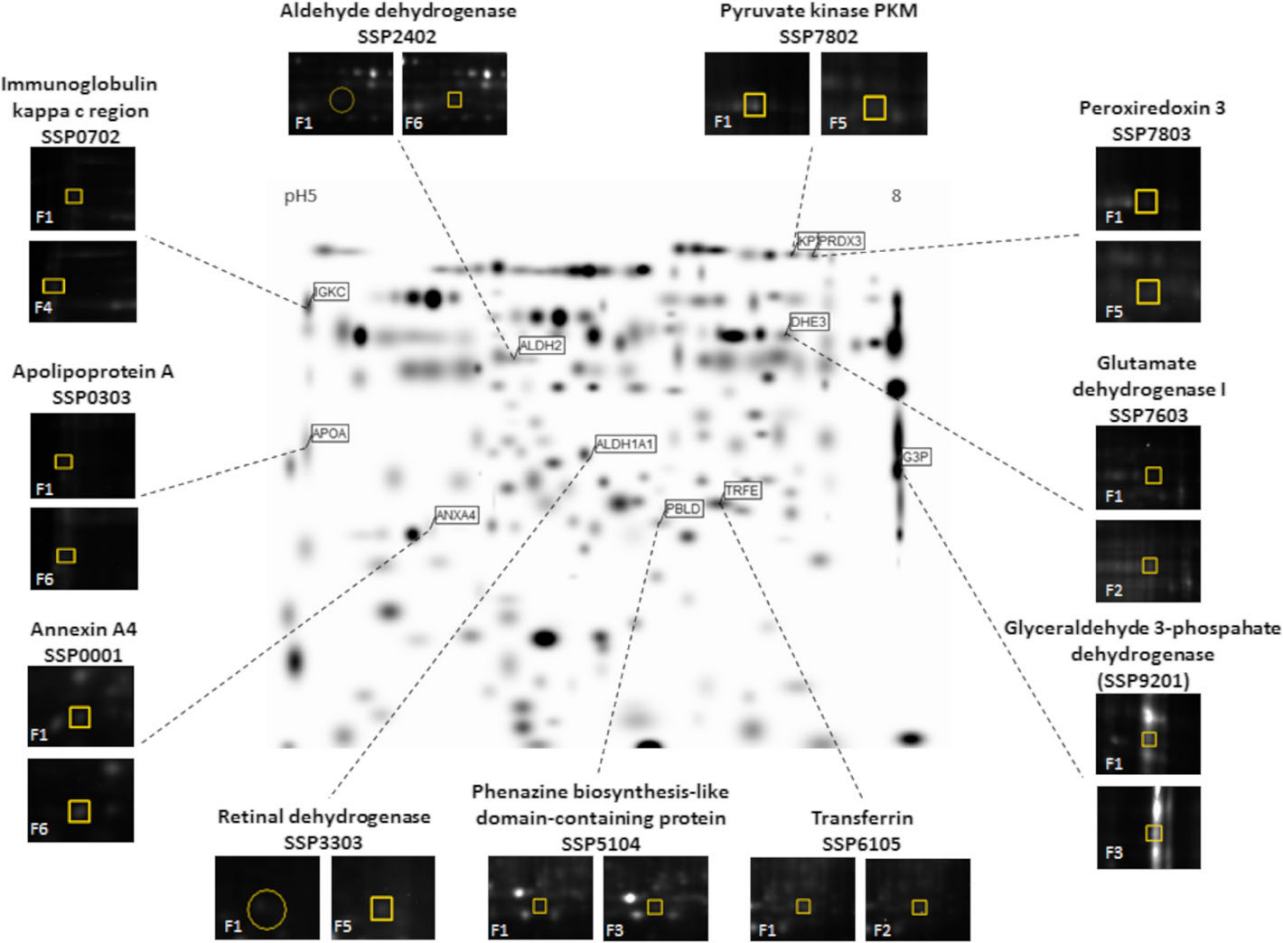
Reference gel image and selected spots. Reference gel image built up from 10 separate 2D-PAGE gels from HBV-infected patient’s liver tissue samples formed by PDQuest, containing 222 valid protein spots. The eleven selected proteins are visualized on reference gel: annexin A4 (SSP0001), apolipoprotein A (SSP0303), immuglobulin kappa chain C region (SSP0702), aldehyde dehydrogenase (SSP2402), retinal dehydrogenase (SSP3303), phenazine biosynthesis-like domain-containing protein (SSP5104), transferrin (SSP6105), glutamate dehydrogenase I (SSP7603), pyruvate kinase PKM (SSP7802), peroxiredoxin 3 (SSP7803), glyceraldehyde 3-phospahate dehydrogenase (SSP9201). The enlarged images display differences in protein expression (spot volume = pixels*spot size) according to F1 stage

Tissue samples from individuals in the same group were pooled together for analysis to flatten intrinsic individual differences and augment common characteristic traits only related to fibrotic stages. Each tissue sample group was labelled with either Cy3 or Cy5 and ran in two different gels (Additional file 1: Figure S1). For tissue samples, ten separate 2D-PAGE gels were semi-automatically matched and resulted in a combined reference image with 222 protein spots (Fig. 1). According to univariate analyses and trend test between individual fibrotic stages 25 spots were obtained and cut from preparative gel for mass fingerprinting (Additional file 1: Figure S2). Mass spectrometry analysis resulted in 12 different protein identities, summarized in Table 2.

**Table 2:** List of identified proteins in tissue samples

Spot No (SSP)	Protein ID	Procession No	PLGS score	Coverage	MW (kDa)	pI	Biological function
0001	annexin A4	P09525	1876	46.7	35860	5.7	epithelial cell differentiation
0303	apolipoprotein A	P02647	2211	46.8	30758	5.4	Carrier (cholesterol)
0702	immuglobulin kappa chain C region	P01834	11563	30.2	11601	5.5	Adaptive immunity
2402	aldehyde dehydrogenase	P05091	4205	46.4	56345	6.7	Alcohol metabolism
3303	alcohol dehydrogenase	P07327	550	22.7	39832	7.9	Alcohol metabolism
5104	phenazine biosynthesis-like domain-containing protein	P30039	1633	32.3	31765	6.1	Epithelial maintenance
6105	transferrin	P02787	922	43.1	77013	6.8	Carrier (Fe)
7603	glutamate dehydrogenase I	P04406	509	17.6	36030	8.7	Glycolysis
7802	pyruvate kinase PKM	P14618	896	25.0	57900	7.8	Glycolysis
7803	peroxiredoxin 3	P30048	804	34.4	27675	7.7	Oxidative stress
9201	glyceraldehyde 3-phospahate dehydrogenase	P04406	509	17.6	36030	8.7	Glycolysis
7804	keratin, type II cytoskeletal 5	P13647	197	15.6	62339	7.8	Cytoskeleton

**Fig. 2:**
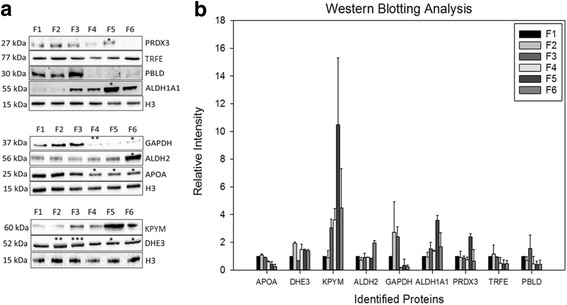
Western blotting analysis of identified proteins from tissue samples. **a** Verification of APOA1, KPYM, GAPDH, DHE3, ALDH2, ALDH1A1, TRFE, PRDX3, PBLD, IGKC, ANX4 by western blotting. Quantification of (**b**) PRDX3, PRDX3, TRFE, ALDH1A1, GAPDH, ALDH2, APOA1, KPYM, DHE3 by ImageLab software v5.2.1 (Bio-Rad). (*) represents significant difference at *p* < 0.05 following student’s *t*-test. (**) represents significant difference at *p* < 0.01, (***) represents significant difference at *p* < 0.001 following student’s *t*-test

Differential expressed proteins were found to be APOA1, KPYM, GAPDH, DHE3, ALDH1, ALDH1A1, TRFE, PRDX3, PBLD, IGKC, ANXA4, KRT5. To confirm stage-dependent changes observed in 2D-DIGE, western blotting analysis was carried out for selected proteins (Fig. 2). The trends of alterations for analyzed proteins were consistent with that of 2D-DIGE results. When each protein expression was compared to that of F1: ALDH1A1 and ALDH2 showed a significant increase in F6 (*p* < 0.05); APOA1 has significantly reduced in F4 and F5 (*p* < 0.01) and F6 (*p* < 0.001); DHE3 is significantly increased in F2 (*p* < 0.01); GAPDH was significantly increased in F3 (*p* < 0.05) and decreased in F6 (*p* < 0.05); PBLD expression is significantly increased in F3 (*p* < 0.05); PKM is significantly increased in F3 (*p* < 0.01) and F4 (*p* < 0.001), also showed an increased trend in F5 and F6; PRDX3 showed an increased trend in F5 and TRFE showed an increased trend in F2 which is consistent with 2D-DIGE results.

### Inter-stage analysis of tissue proteins

**Fig. 3:**
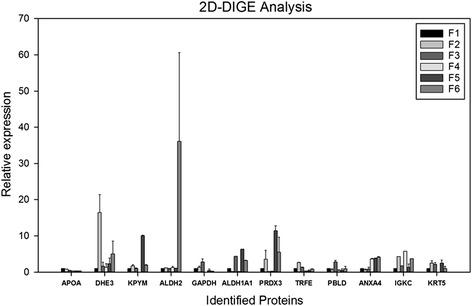
Selected proteins in liver tissue proteome (inter-stage analysis). Protein expression is represented by relative spot volume (pixels*spot size) normalized according to internal standard and are grouped according to Ishak’s classification (F1-F2-F3-F4-F5-F6)

APOA1 (SSP0303) expression was significantly decreased during F3 (*p* < 0.05) and F4, F5, F6 compared to that of F1 (*p* < 0.001) (Fig. 3). Apolipoprotein A, which is included in noninvasive FibroTest is a putative biomarker of HCV associated fibrosis [26]. This well-known biomarker, for the first time with our study, was shown to be downregulated in human liver in a stage specific manner.

DHE3 (SSP7603) expression was significantly increased in F2 compared to that of F1 (*p* < 0.05) (Fig. 3). Pyruvate kinase PKM (KPYM) (SSP7802) expression was significantly increased in F5 as opposed to F1 (*p* < 0.001) (Fig. 3). GAPDH (SSP9201) was significantly increased in F3 (*p* < 0.05) and decreased in F6 (*p* < 0.05) compared to that of F1 (Fig. 3). All of these proteins are members of the glycolysis pathway, justifying the existence of abnormalities in glycolytic mechanism of patients having HBV related liver fibrosis.

ALDH2 (SSP2402) expression was significantly increased in F6 when compared to the results of F1 (*p* < 0.05) (Fig. 3). Aldehyde dehydrogenase is involved in ethanol metabolism along with alcohol dehydrogenase. ADH1A1 (SSP3303) expression was also significantly increased during F3, F5 and F6 (*p* < 0.001) compared to that of F1 (Fig. 3).

TRFE (SSP6105) expression is significantly increased in F2 (*p* < 0.001) and decreased in F4 (*p* < 0.05) when compared to that of F1 (Fig. 3). PRDX3 (SSP7803) expression was significantly increased in F5 and F6 (*p* < 0.001) compared to that of F1 (Fig. 3).

IGKC (SSP0702) was significantly increased during F2, F4 and F6 (*p* < 0.001) compared to that of F1 (Fig. 3). PBLD (SSP5104) expression is significantly increased in F3 compared to that of F1 (*p* < 0.05) (Fig. 3). Altered expression of these inflammatory proteins, IGKC and PBLD, depends on occasionally fluctuating inflammatory activity in liver due to fibrosis and HBV infection; hence, this situation may not be directly associated with patient’s fibrotic stages.

ANXA4 (SSP0001) expression was significantly increased during F4 (*p* < 0.01), F5 (*p* < 0.001) and F6 (*p* < 0.01) compared to that of F1 (Fig. 3). KRT5 (SSP7804) expression was significantly increased during F2 (*p* < 0.05) compared to that of F1 (Fig. 3).

### Associations between identified proteins and cellular pathways in liver

**Fig. 4:**
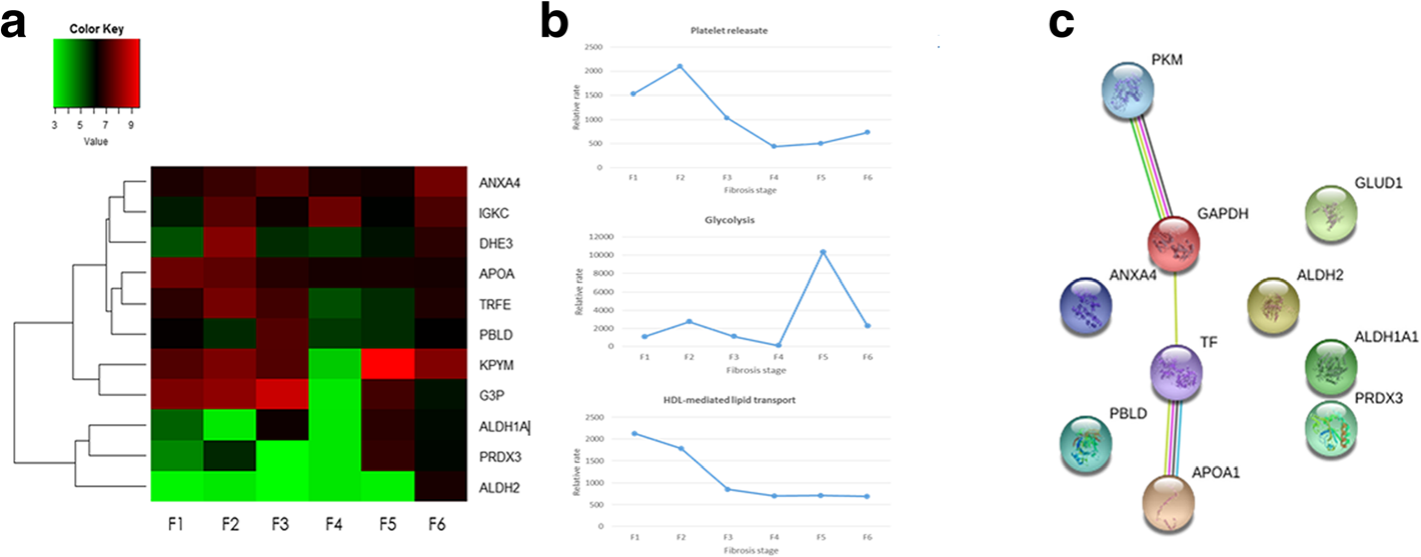
Bioinformatic analysis. **a** Clustering of identified proteins from tissue samples according to tissue fibrotic stages. The degree of similarity in profiles is shown by colour (base-two logarithmical scale is above the figure). The average protein abundance of the 11 identified proteins (labelled with Cy3 and Cy5 dyes) is calculated relative to internal standard (labelled with Cy2 dye). **b** Alterations in identified cellular pathways along with fibrosis progression. Alterations in pathways were determined by using expression analysis of Reactome database. **c** Evidence view of identified liver tissue proteins in String database. (Confidence score > 0.700). Different line colors represent the types of evidence for the association. *Green* indicates neighborhood. *Gray* indicates co-expression. *Cyan* indicates association in current databases. *Magenta* indicates experimental data. *Yellow* indicates text mining

A heat map graphic was created to classify proteins that share the same expressional alterations and cluster together (Fig. 4a). We observed two main clusters that include sub-clusters. First cluster has three sub-clusters comprising first with ANXA4 and IGKC partnering and having DHE3 as a neighbor; second with APOA1 and TRFE together, also having DHE3 as a neighbor; third with KPYM and GAPDH. The second cluster includes ALDH1A1 having two separate sub-clusters with PRDX3 and ALDH2, respectively. From this heat map, we can observe close association between APOA1 and TRFE, clustering of PRDX3 with ethanol metabolism proteins, ALDH1 and ALDH2, suggesting the role of PRDX3 in alcohol metabolism in HBV-associated liver fibrosis.

For the prediction of possible involvement of the identified proteins in the fibrosis development process in HBV infection and thus reveal the pathways influenced by HBV related fibrosis pathogenesis, we processed the protein dataset in EnrichNet. Enrichment analysis using KEGG and Reactome databases exposed implications of the identified proteins in several cellular pathways (Additional file 1: Table S2 and S3). XD-scores were regarded to be more than 0.49 and 0.80 threshold values, as estimated by the application for the KEGG and Reactome databases, respectively.

To further investigate the alterations in these identified pathways (further platelet release, glycolysis, HDL mediated lipid transport) along with fibrosis progression, the rate trend of these pathways were assessed by Reactome expression analysis (Fig. 4b). This analysis showed a decreasing trend in further platelet release along with liver fibrosis. Glycolysis showed fluctuations along with the fibrosis development. HDL mediated lipid transport was determined by APOA1 levels. As APOA1 levels were decreasing toward cirrhosis, HDL mediated lipid transport activity was also showed a decreasing trend toward cirrhosis.

Highest confident string analysis (confidence score > 0.9), showed a functional link between PKM and GAPDH (combined confidence score = 0.946), TRFE and APOA11 (combined confidence score = 0.957), TRFE and GAPDH (confidence score = 0.941) (Fig. 4c). Evidence suggest that the co-expression of PKM and GAPDH are not yet shown in *Homo sapiens* but they are shown to be co-expressed in homologues species closest to *Homo sapiens* (Additional file 1: Table S4). Experimental research showed an altered expression level in HCC for PKM and GAPDH [27] and also revealed an intact protein-protein interaction between PKM and GAPDH in nonmalignant skin cancer [28], suggesting the importance of this interaction in tumor supression and apoptosis.

Evidence suggest that the co-expression of TRFE and APOA1 are not yet shown in *Homo sapiens* but they are shown to be co-expressed in close homologues species (Additional file 1: Table S5). Experimental researches also showed an intact protein-protein interaction between TRFE and APOA1 in human sera [29]. TRFE and APOA1 interaction is also present in “Release of platelet secretory granule components” pathway as found out in our Reactome database research.

We also performed string analysis with high confidence (0.900) to all proteins that come up from inter-stage analysis of tissue samples to search for specific interacting partners that can be related with liver fibrosis. As a result, we observed that there is a significant link between PKM and HIF-1α which is an important interacting partner of Hepatitis virus X protein (HBx) (Additional file 1: Figure S3).

## Conclusions

Liver fibrosis currently represents one of the severe global health problems and chronic HBV infection is one of the major risk factors of hepatic fibrogenesis. Although, liver biopsy is currently accepted as the gold standard for diagnosis of liver fibrosis, the tissue specific biomarkers would be invaluable to overcome inter-observer variability and to offer an unbiased clinical result. The first step in the development of such biomarkers is the knowledge of disease-associated proteins and pathways. Although various proteomic studies that investigated altered protein levels in HCV infected liver tissue samples [13, 18, 30] are present in literature, there are no available study concerning proteomic changes in HBV infected tissue samples.

In this study, we identified cellular and metabolic pathways that are associated with liver fibrosis in HBV infection to highlight critical players in the development of fibrosis. Major cellular pathways like glycolysis, alcohol metabolism, oxidative stress regulation, platelet release and HDL mediated lipid transport get affected by HBV infection. Apart from these observed effects on specific pathways, some individual protein expression alterations were observed. Changes in these protein levels can also play a critical role in various cellular pathways, especially in cell motility, immunity, cellular transport, alcohol metabolism and oxidative stress, mediating stage dependent liver fibrogenesis.

One of the affected mechanisms in liver fibrosis is cell motility, and our results demonstrated also a change in this mechanism by revealing enhanced expression of Keratin 5, which is member of intermediate filaments constitute the cytoskeletal scaffold within epithelial cells, assists cell architecture and provides the cells with the ability to withstand mechanical and non-mechanical stresses [31]. During fibrosis, epidermal growth factor (EGF) expression in the liver increases and the activation of epidermal growth factor receptor (EGFR) by increased EGF levels results in upregulation of KRT5 [32]. ANXA4 which is responsible for membrane fusion of exocytotic vesicles was upregulated in our study. There are body of evidence suggesting an increase in ANXA4 expression in terminally differentiated hepatocytes and HCC [33]. Therefore, increased ANXA4 expression at advanced fibrosis stages might be an indicator of ongoing carcinogenesis. Altogether, these alterations may suggest the role of cell motility in liver fibrosis.

Observation of transferrin level changes in our study, highlight the importance of iron metabolism in cirrhosis since transferrin produced by hepatocytes is a crucial member of iron metabolism [34]. A recent study demonstrated a significant decrease in serum transferrin levels in advanced fibrosis than in mild fibrosis [35]. In combination with our findings, it is tempting to propose that transferrin synthesis might increase during the early stage of liver disease, and thereafter it decreases as fibrosis advances as a result of reduced capacity of transferrin synthesis.

We also observed a downregulation in APOA1 protein that plays a role in HDL mediated lipid transport. The relation of HDL mediated lipid transport with liver fibrosis was well-known and noninvasive FibroTest involving serum apolipoprotein-A1 have already been in clinical use for diagnosis of liver fibrosis [26]. APOA1 which is the carrier of (HDL), is secreted by hepatocytes and was shown to carry anti-inflammatory properties [36]. Experiments have shown that the level of serum APOA1 was downregulated in CHB and CHB-related HCC [37, 38]. There are studies revealing deregulation of lipid metabolism and oxidative stress in HCV infected fibrotic liver tissue samples [18]. Increased ROS levels and dysregulated hepatic lipid metabolism, altogether, causes lipotoxicity leading to more and more ROS accumulation and eventually induction of hepatocyte apoptosis and fibrogenesis [39]. In our study, we also showed similar dysfunctioning in lipid metabolism and oxidative stress in HBV infected liver tissues.

**Fig. 5:**
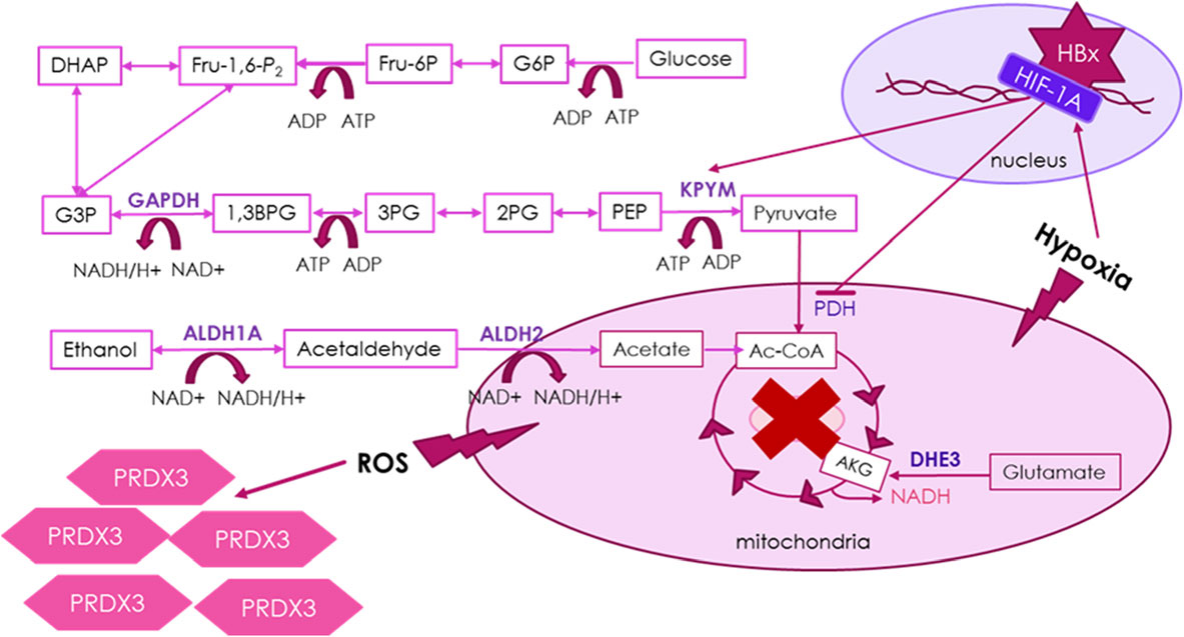
Proposed model for HBV related liver fibrosis. HBx interacts with HIF-1A that activates PKM expresstion and inhibits PDH expression. This causes and alternate route for Ac-CoA production that results in increased ALDH1A1 and ALDH2 expression and also ROS accumulation, leading to elevated PRDX3 level

Liver is known to be as a central organ in lipogenesis, gluconeogenesis and cholesterol metabolism. Therefore, alterations in insulin response, β-oxidation, lipid storage and transport, autophagy and an imbalance in chemokines and nuclear receptor signaling are all responsible for liver fibrogenesis [39]. In our study, we identified glycolysis as an altered pathway in HBV associated liver fibrosis due to changes in the DHE3, GAPDH and KPYM expression and also demonstrated altered expression of ALDH2 and ALDH1A1 which are the key members of alcohol metabolism contributing Acetyl Coenzyme A (Ac-CoA) production and ROS accumulation. Recent data has demonstrated that the transdifferentiation of quiescent HSCs into myofibroblasts activates glycolysis and results in lactate accumulation through the induction of HIF1-α for metabolic reprogramming of HSCs [40]. One of the main contributors in HBV infection is HBx protein that has been shown to interact with various host proteins [41, 42]. HIF-1α which is one of the interaction partners of HBx [43], activates the transcription of PKM [44]. HIF-1α also trans-activates the gene encoding pyruvate dehydrogenase kinase 1 (PDK1) which inactivates the TCA cycle enzyme, pyruvate dehydrogenase (PDH) responsible for conversion of pyruvate to acetyl-CoA thereby inhibiting TCA cycle [45]. We observed an increase in PKM expression in advanced fibrosis and cirrhosis, probably due to increased HIF-1α production. Moreover, we observed increased ALDH2 and ADH1A1 expression which are the enzymes responsible for metabolizing alcohol to acetate in cirrhosis. Therefore, we propose a model in which inhibition of PDH by HIF-1α triggers an alternative method for acetyl-coA production which is in our case through alcohol metabolism. However, upregulation of this pathway along with decreased APOA1 levels disrupts hepatic lipid metabolism and results in ROS accumulation. Increased ROS accumulation therefore induces peroxiredoxins which are responsible for reducing intracellular H_2_O_2_, and it can be the reason of increased PRDX3 levels observed in our study. As a result, observed activation of PKM and possible inhibition of PDH by HIF-1α may upregulate glycolysis pathway to overcome the TCA cycle inhibition (Fig. 5).

It is worthy to mention that all samples that were used in this study were needle biopsies that can only represent a very small fraction of the liver (approximately 1/50000). Since fibrotic tissue is not distributed homogeneously inside the organ, this form of liver biopsy may bring on sampling error [46]. By taking these facts into account, in our study, we pooled the samples from each stage to minimize the sampling errors resulting from biopsy procedures.

In conclusion, our proteomic approach characterized various pathways associated with HBV infection dependent-stage specific liver fibrosis. Our results suggest HIF-1α, through its interactions with HBx, as a critical modulator of HBV associated liver fibrosis, yielding variations mainly in glycolysis, oxidative stress, ethanol and lipid metabolism within the hepatic cells causing a fibrotic environment by the rearrangement of extra cellular matrix. The study of Moon et al. showed reduced liver fibrosis in HIF-1α deficient mice and therefore pointed HIF-1α as a crucial regulator of profibrotic mediator produced during fibrogenesis, correlating well with our findings [47].

For future studies, validation of determined pathways from our study in large datasets and multiple cohorts would further allow the usage of protein candidates as diagnostic and therapeutic targets in HBV-related fibrosis.

## Additional file

Additional file 1: Supplementary Information. (DOCX 2221 kb)
