# Supplementary material for: Proteomic profiling of HBV infected liver biopsies with different fibrotic stages
Source: Proteome Sci. 2017 Apr 20;15:7. doi: 10.1186/s12953-017-0114-4 (PMC5399407; doi:10.1186/s12953-017-0114-4)
Supplement: Additional file 1: — Supplementary Information. (DOCX 2221 kb) [file 12953_2017_114_MOESM1_ESM.docx]

**Additional file 1**

**
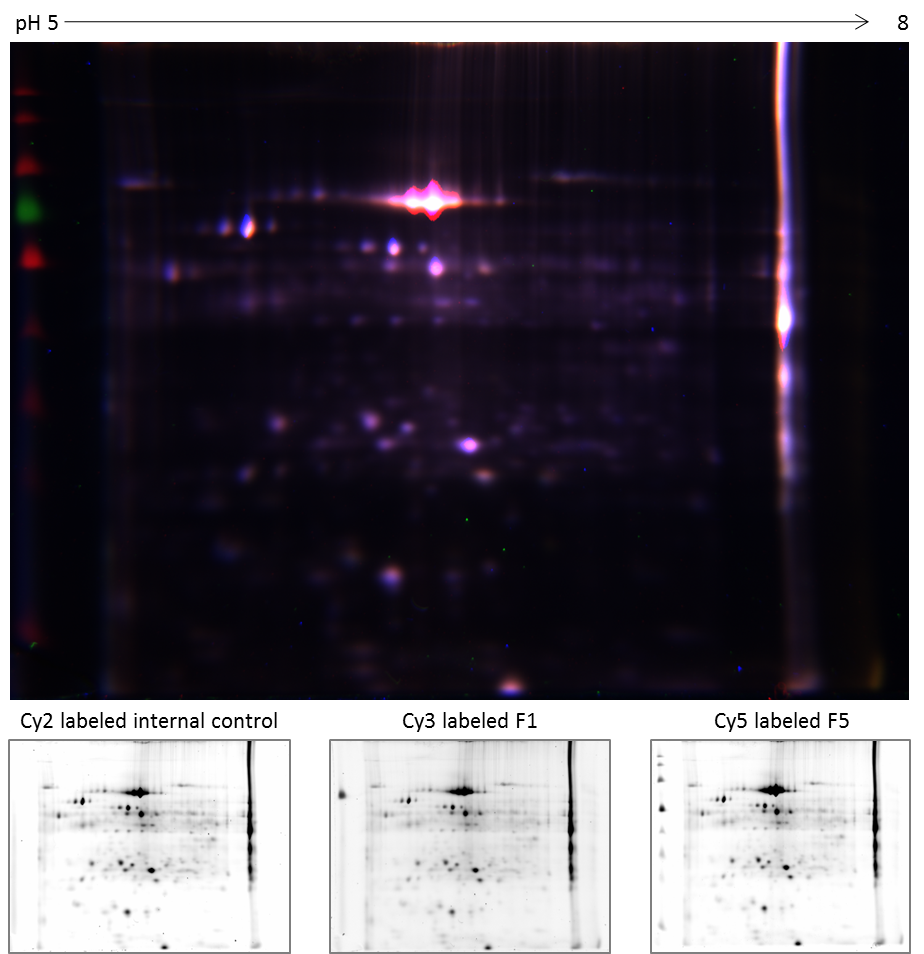
**

**Fig. S1 DIGE experiment.** Representative picture of DIGE gels of tissue samples. On the top overlay of three dye scan-images; on the left, the scan of Cy2 of internal standard; on the center, the scan-image Cy3 of F1 pool; on the right, the scan-image Cy5 of F5 pool.

**
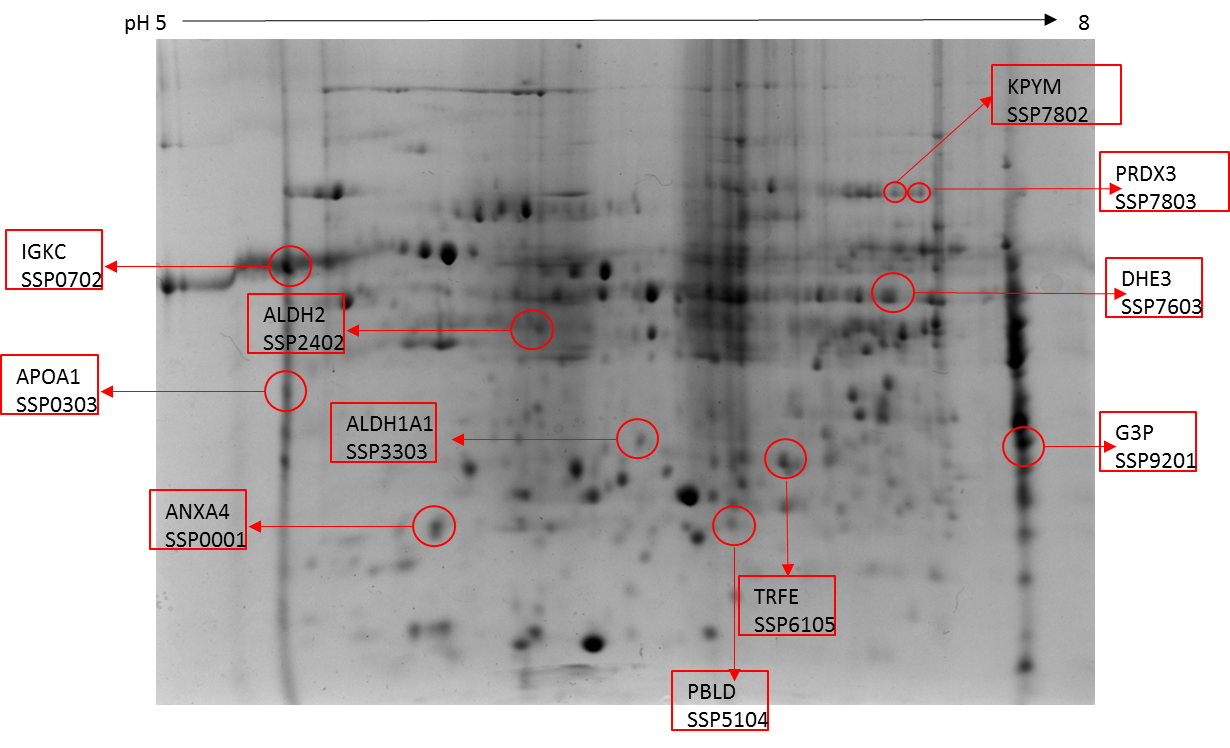
Fig. S2 Preparative gel image of tissue samples.** The 12% polyacrylamide gel has a linear pH5-8 range. A total of 12 spots were differentially expressed followed univariate analyses and identified by mass spectrometry are marked by circles and their spot number (SSP).

**
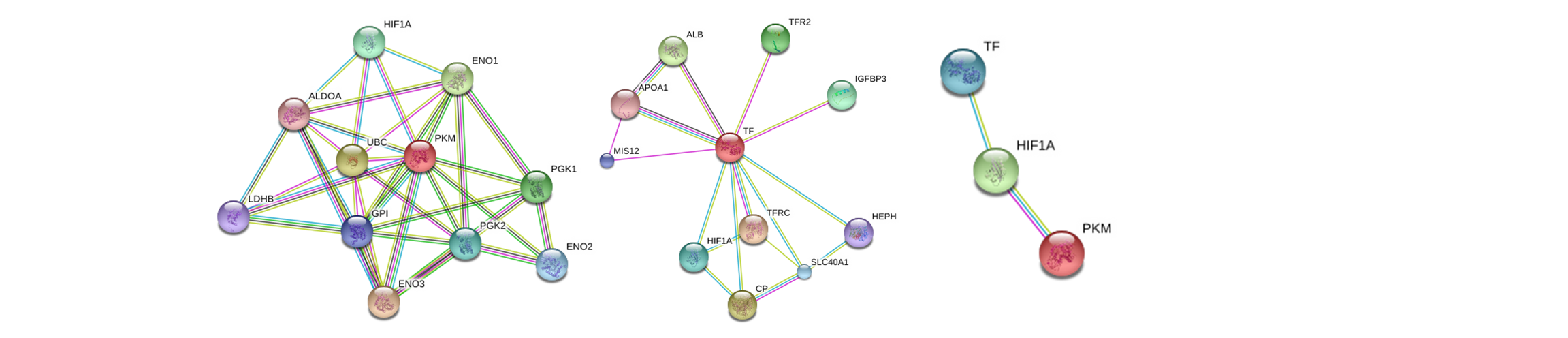
**

**Fig. S3** **Evidence view of identified liver tissue proteins in String database.** (Confidence score > 0.900). **(A)** String analysis of PKM revaling its association with HIF-1α. **(B)** String analysis of PKM revaling its association with HIF-1α. **(C)** Evidence view of PKM, TF and HIF-1α together.

**Table S1 Patient’s demographic data according to fibrotic stages.**

|  | F1 (n=7) | F2 (n=20) | F3 (n=12) | F4 (n=3) | F5 (n=2) | F6 (n=3) |
| --- | --- | --- | --- | --- | --- | --- |
| Sex |  |  |  |  |  |  |
| Male | 4 | 9 | 9 | 3 | 2 | 3 |
| Female | 3 | 11 | 3 | 0 | 0 | 0 |
| Age | 40 (32-55) | 39.2 (19-65) | 39.4 (20-66) | 52 (46-59) | 49 (48-50) | 49.3 (35-59) |
| ALT (U/L) | 43.3  (14-75) | 102.0  (21-441) | 107.6  (29-439) | 70.0  (52-82) | 79.5  (33-126) | 64.7  (32-125) |
| AST (U/L) | 28.0  (17-46) | 59.8  (21-182) | 66.3  (23-309) | 55.3  (39-66) | 44.0  (26-62) | 45.7  (35-64) |
| PLT (x10^3^/µL) | 238  (170-301) | 235  (161-370) | 203  (146-301) | 180  (170-193) | 186  (148-224) | 185  (119-291) |
| PT (sec) | 13.0  (12-14) | 13.1  (12.4-14.3) | 13.4  (12.1-16.1) | 13.3  (12,3-13,9) | 13.4  (12.3-13.4) | 14.3  (13.6-14.7) |
| INR | 1.00  (0.87-1.13) | 1.01  (0.85-1.25) | 1.03  (0.90-1.15) | 1.05  (0.93-1.21) | 0.96  (0.9-1.02) | 1.24  (1.15-1.26) |
| APTT (sec) | 30.9  (27.5-34.0) | 30.4  (26.5-35.4) | 31.4  (27.2-40.06) | 30.2  (29.4-30.6) | 30.0  (29.8-30.1) | 31.0  (30.2-32.1) |
| HBV DNA (x10^5^ IU/L) | 0.34  (0.08-0.56) | 902.00 (8728-0.03) | 392.64  (0.08-444) | 624.25  (4.50-1700) | 10.06  (2.77-17.34) | 30.17  (0.001-90.48) |
| Hai | 2.7 (2-4) | 5.2 (2-10) | 5.3 (2-9) | 8.7 (7-10) | 6.0 (5-7) | 9.3 (8-11) |
| HDL | 42 (27-50) | 48 (32-67) | 49 (28-72) | 49 (33-65) | 39 (39-39) | 38 (36-40) |
| LDL | 112 (61-144) | 115 (80-167) | 115 (86-157) | 126 (105-146) | 125 (125-125) | 124 (89-158) |
| Triglyseride | 70 (50-87) | 87 (60-161) | 141 (59-359) | 125 (91-159) | NA | 96 (65-125) |
| Glucose | 114 (86-256) | 95 (76-145) | 112 (82-295) | 102 (88-116) | 101 (90-112) | 150 (83-217) |
| Waist (cm) | 89 (74-100) | 92 (62-110) | 91 (69-114) | 95 (95-95) | 106 (91-120) | 99 (72-116) |
| BMI | 26 (20-34) | 27 (19-38) | 27 (20-34) | 27 (27-27) | 27 (23-30) | 29 (21-35) |

**Table S2 Functional association of the identified protein dataset with KEGG cellular pathways.**

| **Annotation (pathway/process)** | **XD-score*** | **Fisher q-value** |
| --- | --- | --- |
| Glycolysis / Gluconeogenesis | 0.56 | 1.71E+09 |

* XD-score significance threshold: (reqression fit equivalent to Fisher q-value of 0.05 + upper bound of 95% confidence interval for linear fitting) = 0.49

**Table S3 Functional association of the identified protein dataset with Reactome cellular pathways.**

| **Annotation (pathway/process)** | **XD-score*** | **Fisher q-value** |
| --- | --- | --- |
| Further platelet releasate | 0.84 | 0.09 |
| Glycolysis | 0.80 | 0.09 |
| HDL mediated lipid transport | 0.80 | 0.88 |

* XD-score significance threshold: (reqression fit equivalent to Fisher q-value of 0.05 + upper bound of 95% confidence interval for linear fitting) = 0.8

**Table S4 Evidence suggesting a functional link between PKM and GAPDH.**

| Neighborhood in the Genome | Homologous genes are neighbors in other genomes (score 0.284) |
| --- | --- |
| Co-Expression | Putative homologs are coexpressed in other species such as *Rattus norvegicus, Mus musculus, Gallus gallus, Macaca mulatta* (score 0.474). |
| Experimental/Biochemical Data | Intact protein-protein interaction (score 0.644) |

**Table S5 Evidence suggesting a functional link between TF and APOA.**

| Co-Expression | Putative homologs are coexpressed in other species such as *Danio rerio and Mus musculus* (score 0.071). |
| --- | --- |
| Experimental/Biochemical Data | Intact protein-protein interaction (score 0.644) |
| Association in Curated Databases | “Release of platelet secretory granule components” pathway (score 0.900) |
